# Ethnobotanical dataset on local edible fruits in North Sulawesi, Indonesia

**DOI:** 10.1016/j.dib.2019.104681

**Published:** 2019-10-18

**Authors:** Trina Ekawati Tallei, Johanis Jullian Pelealu, Hard Napoleon Pollo, Gracia Alice Victoria Pollo, Ahmad Akroman Adam, Yunus Effendi, Agung Karuniawan, Souvia Rahimah, Rinaldi Idroes

**Affiliations:** a Department of Biology, Faculty of Mathematics and Natural Sciences, Universitas Sam Ratulangi, North Sulawesi, Indonesia; b Forestry Study Program, Faculty of Agriculture, Universitas Sam Ratulangi, North Sulawesi, Indonesia; c Biology Master Study Program, Faculty of Biology, Universitas Gadjah Mada, Yogyakarta, Indonesia; d Dentistry Study Program, Faculty of Medicine, Universitas Sam Ratulangi, North Sulawesi, Indonesia; e Biology Study Program, Faculty of Science and Technology, Universitas Al Azhar, Jakarta, Indonesia; f Department of Agronomy, Faculty of Agriculture/Graduate School, Universitas Padjadjaran, Bandung, Indonesia; g Faculty of Agro-Industrial Technology, Universitas Padjadjaran, Bandung, Indonesia; h Department of Pharmacy, Faculty of Mathematics and Natural Sciences, Universitas Syiah Kuala, Banda Aceh, Indonesia

**Keywords:** Biodiversity, Ethnobotany, Local fruit, North sulawesi, Wallacea

## Abstract

This dataset describes the knowledge of local people in North Sulawesi on local edible fruits which can be eaten raw or used as medicine. North Sulawesi is located in the Wallacea zone [1,2] and has a high biodiversity of local fruits that are not yet fully exploited. Fruits are available as rich sources of vitamins, fibres, minerals, and phytochemicals [3] for local people's diet and health. Ethnobotany was used to collect data for the documentation of local knowledge on the existence, the use, and conservation practices of local fruits using semi-structured and structured interviews and questionnaire. There were 27 recorded families of local edible fruits, predominated by Myrtaceae and Anacardiaceae. Some fruits were found abundantly, but some were rarely found, especially those which were endemic to North Sulawesi. The fruit trees were mostly self-grown, and the fruits were eaten by the community themselves. In general, they were well aware of the types of local fruits that could be eaten raw. Knowledge of local fruits were passed on from generation to generation. Most people claimed that local fruits which could be eaten raw were also used for medicine and maintaining health. Most of the local fruits used as medicines were not made as medicinal preparations, but eaten raw or cooked. However, most people did not know exactly about the efficacy of the fruits. Types of diseases that were claimed to be cured by using local fruit among others were sprue, high cholesterol and digestive disorders. The possibility of future youth generations to consume these fruits was very high, according to most people. But they were worried that the younger generation in the future would prefer imported fruits. The community in general knew that these local fruits needed to be conserved, but they did not yet know how to maintain the existence of these local fruits in the future, apart from their current practices.

**Table:** Specifications Table

Subject area	*Ethnobotany*
More specific subject area	*Edible local fruits, medicinal use of local fruits*
Type of data	*Table, graph*
How data was acquired	*Questionnaires and personal interviews on local people's knowledge and perceptions of local fruits and their conservation practices. The lists of questionnaire and interview are available at DOI:*https://doi.org/10.17632/h4z52vfhmc.1#folder-f3f8a5a4-e0d7-4c05-8432-a3a55e22b635
Data format	*Analyzed* (*the raw dataset is available at DOI:*http://dx.doi.org/10.17632/tgwfm7hhkr.3#folder-872d9519-5106-455f-ab9f-5fe88b6b3f4a)
Experimental factors	*Development of questionnaires and list of material for the interview*
Experimental features	*The use, source, and conservation effort of the edible local fruits*
Data source location	*Six regencies in North Sulawesi Indonesia*
Data accessibility	*Data are included in this article.*

**Table:** 

**Value of the Data** • The data can be regarded as base line information about the local edible fruits which can be eaten raw or used as medicine. • The data is very useful for conservationists of local fruits that can be eaten raw and/or used as basic ingredients for traditional medicines. • The data can be used as a reference to find out how far the knowledge of local fruits is known by local communities in order to maintain the sustainability of these fruits. • The data can be used as a reference for comparative studies of fruits that can be used as traditional medicines and the possibility to be studied further about the bioactive content of these fruits. • The data provide information on biodiversity of ethnomedicinal local edible fruits in these areas which are economic importance for indigenous communities, natural conservation, and educational material.

## 1 Data

**Fig. 1:**
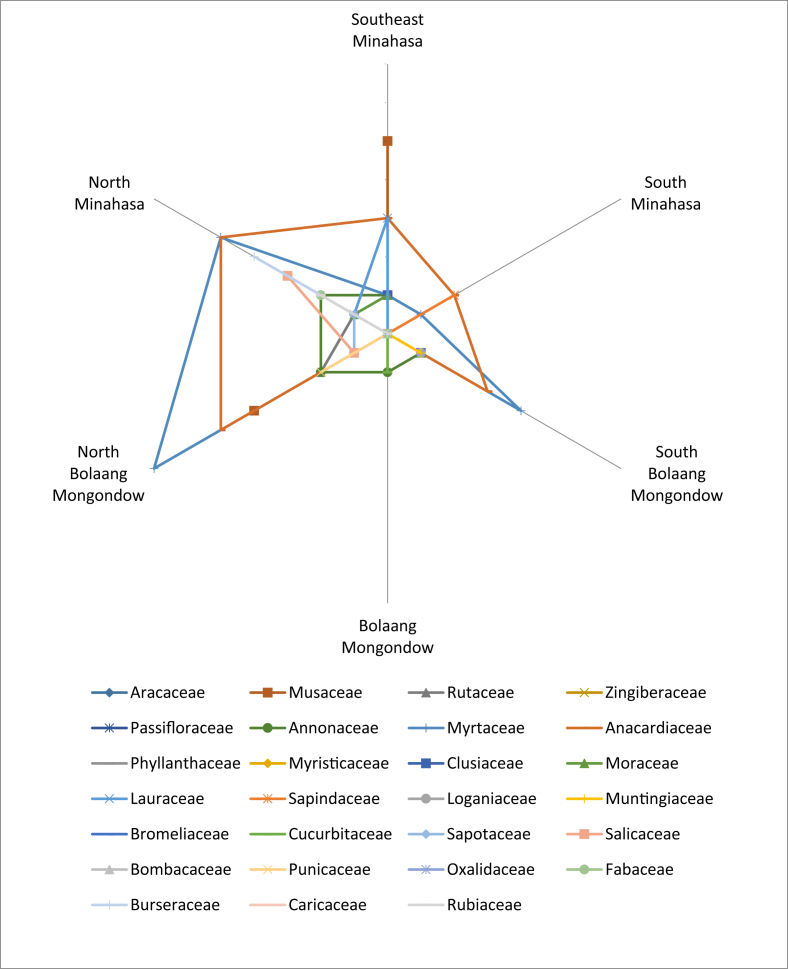
Observed families of local edible fruits distributed in six regencies in North Sulawesi.

The dataset presents an ethnobotanical study on local knowledge on the existence, the use, and the conservation practices of local edible fruits (the raw dataset is available at DOI: http://dx.doi.org/10.17632/tgwfm7hhkr.3#folder-872d9519-5106-455f-ab9f-5fe88b6b3f4a). Fig. 1 shows the data on edible fruit families, which were dominated by Myrtaceae and Anacardiaceae. Predominating families in each regency can be described as follows: Musaceae in Southeast Minahasa, Myrtaceae and Anacardiaceae in North Minahasa, Myrtaceae, Anacardiaceae, and Musaceae in North Bolaang Mongondow, Myrtaceae in South Minahasa, and Muntingiaceae, Anacardiaceae, and Myrtaceae in South Bolaang Mongondow. North Minahasa and North Bolaang Mongondow had the most number of families of edible local fruits.

**Fig. 2:**
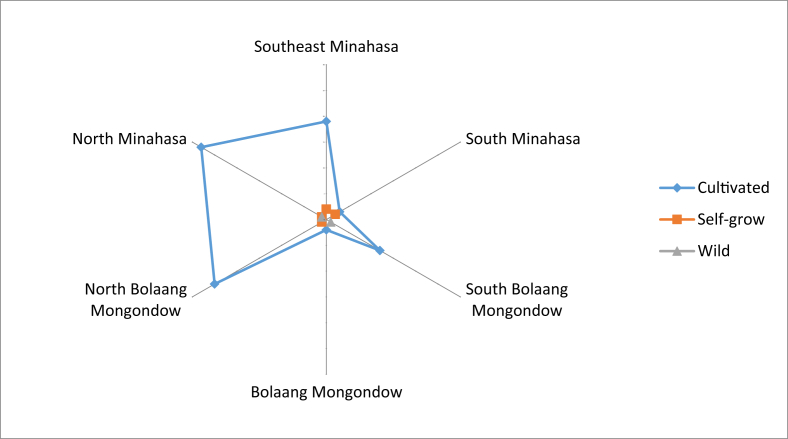
The growth status of the fruit plants.

**Fig. 3:**
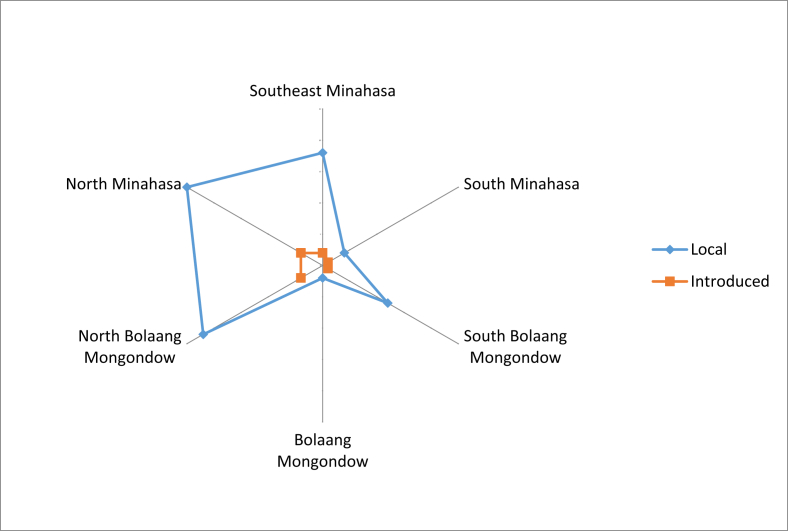
The status of the fruits.

**Fig. 4:**
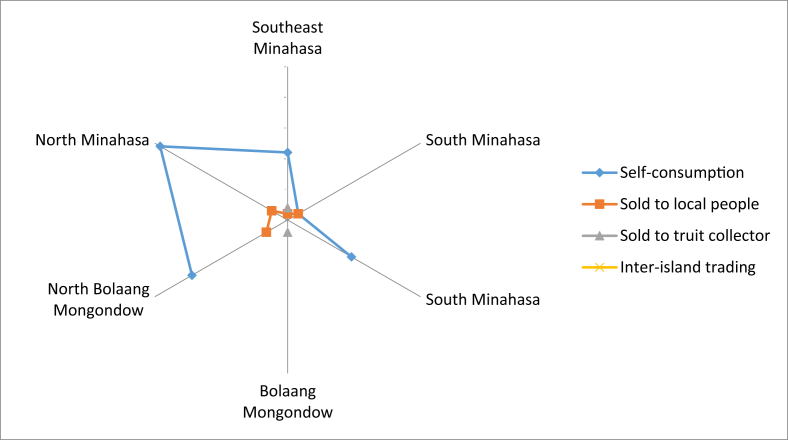
The status of the consumption of the edible local fruits.

**Fig. 5:**
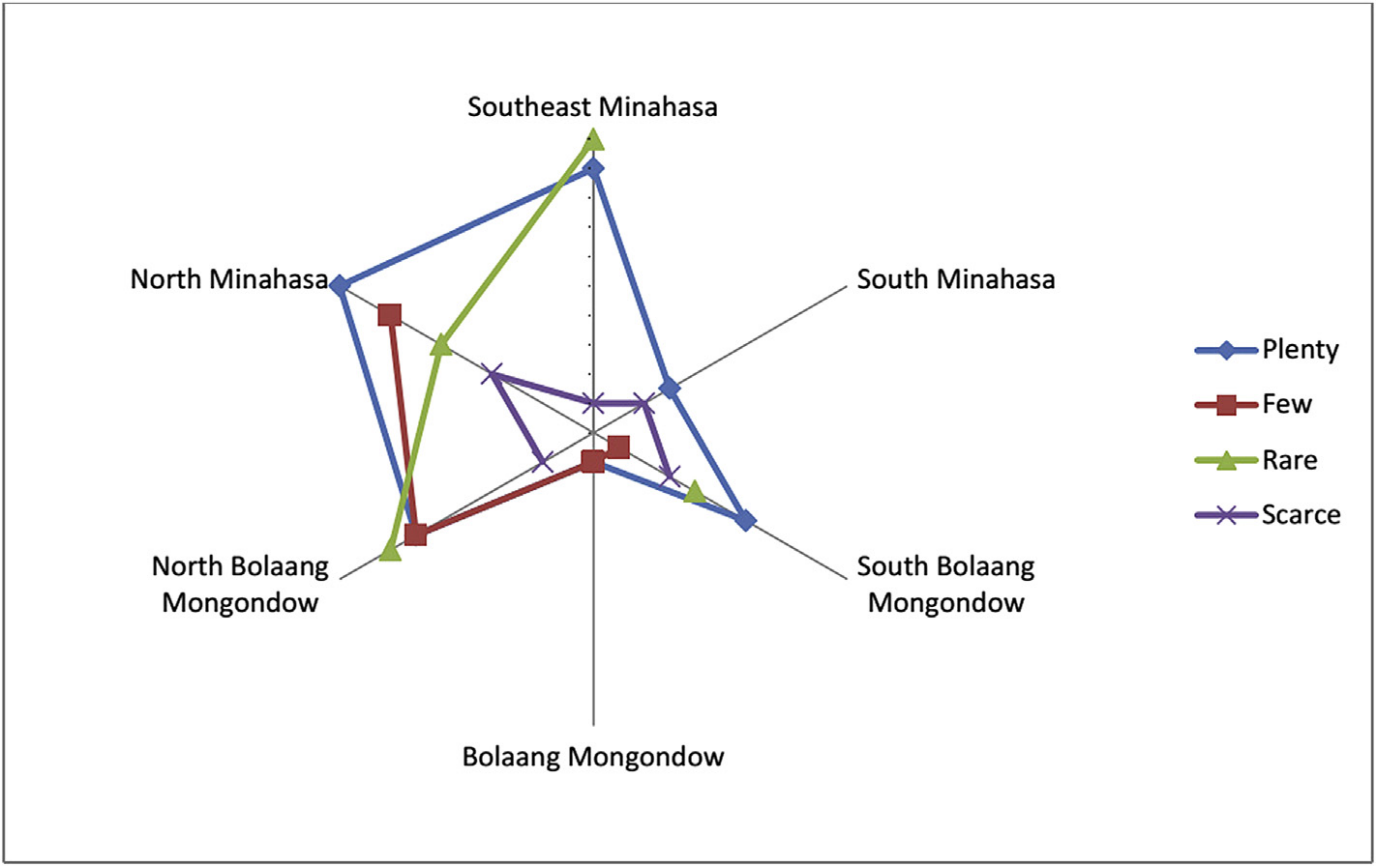
Availability of the fruits locally.

**Fig. 6:**
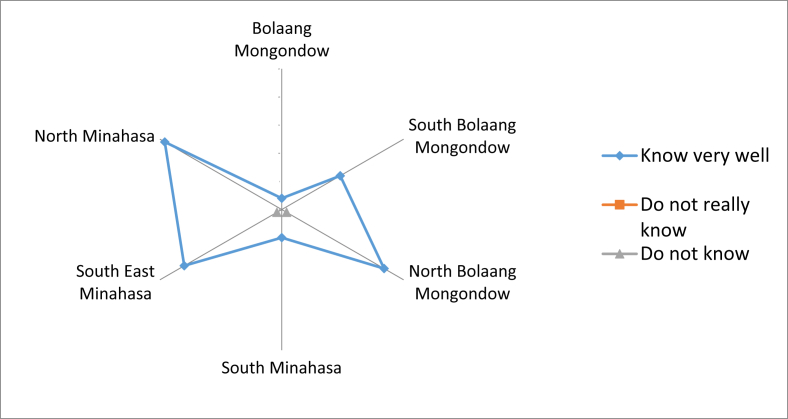
Local community knowledge on local edible fruits which can be eaten raw, both cultivated and grown wild in the yard or forest.

The growth status of these fruits are shown in Fig. 2. Most of the fruit plants were cultivated. Most of the fruit obtained in location was said to be local fruits, and only a small portion was introduced (Fig. 3). The fruits were mostly consumed by the locals, whereas only small numbers were sold to others and fruit collectors (Fig. 4). Some fruits were abundantly available in most of the regencies, but there were some fruits that were already scarcely found (Fig. 5), especially those that were endemic to North Sulawesi and their tastes were less pleasing. The locals generally had knowledge on the kind of edible local fruits that could be eaten raw. Only small amount of them knew less. Some of the fruits could be obtained easily because they were cultivated, as well as grown wild in the yard or forest (Fig. 6).

**Fig. 7:**
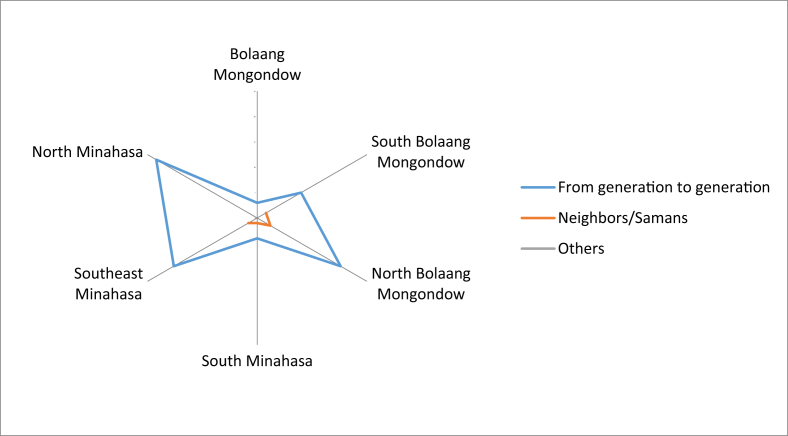
First time receiving knowledge on local edible fruits.

**Fig. 8:**
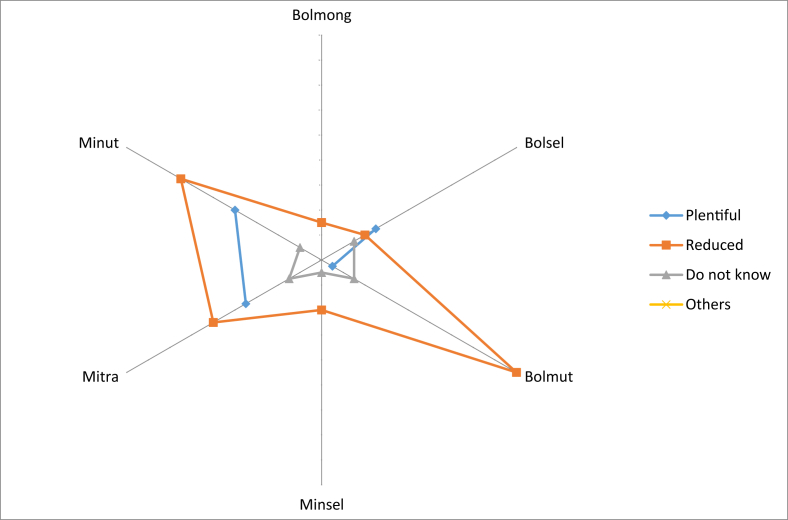
Knowledge of the number of edible fruits obtained from the forest.

The knowledge on the edible local fruits was passed on from generation to generation. It was also obtained from neighbors because there were also people who were married to the local people, so they could get that knowledge (Fig. 7). The fruits could be accessed easily from their own agriculture land, yard, and forest. Most people claimed that the local fruit species that could be eaten raw and found in the forest had decreased. Some communities stated that there were still many supplies, but a small number did not know the availability of local fruit in the forest. Based on comparisons between regencies, many types of fruit that had been reduced were found in North Bolaang Mongondow. The most abundant supply of fruit species in the forest was in North Minahasa (Fig. 8).

**Fig. 9:**
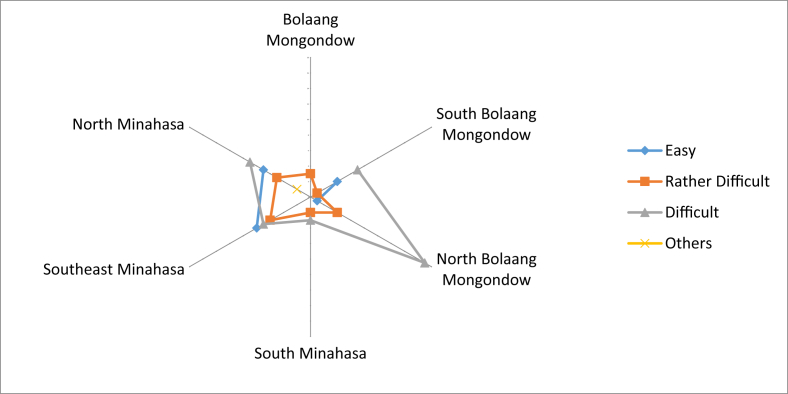
Access to get local fruits that could be eaten raw from the forest.

**Fig. 10:**
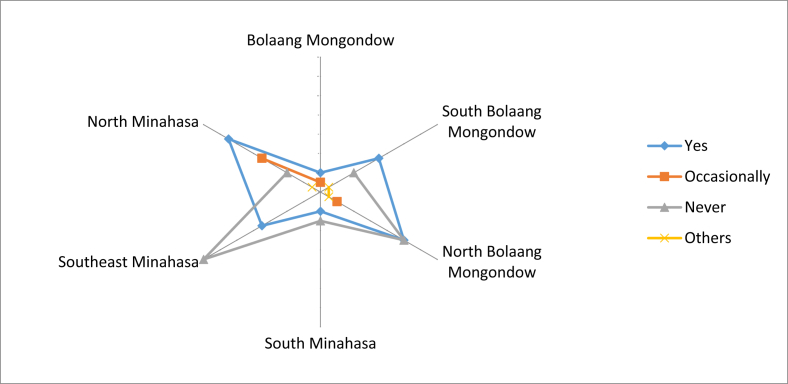
The use of local fruits that can be eaten raw as medicine and for maintaining health.

The ease of access to obtain this type of local fruit varied greatly in each regency. Most people stated that access was difficult, especially in North Bolaang Mongondow. Communities who stated that access was rather difficult and some which were easily obtained were from respondents in Southeast Minahasa and North Minahasa (Fig. 9). Most people in North Minahasa, Southeast Minahasa, South Bolaang Mongondow, and Bolaang Mongondow claimed that local fruits which could be eaten raw were also used as medicinal materials and to maintain health. Most respondents from Southeast Minahasa and North Bolaang Mongondow did not use the fruits for medicinal purposes (Fig. 10).

**Fig. 11:**
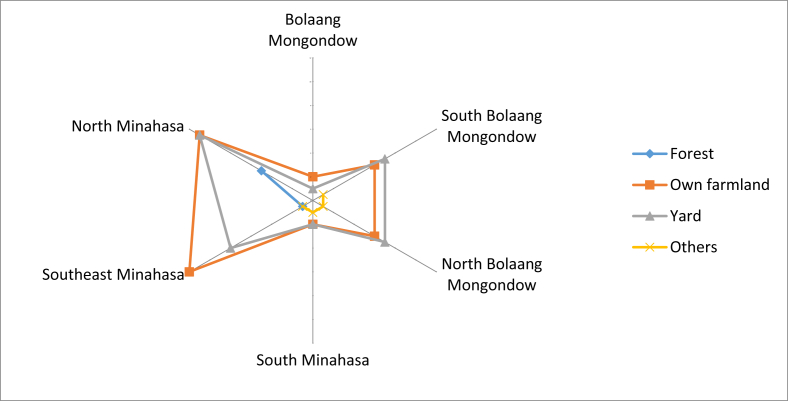
Locations of the local edible fruits.

These local fruits were often obtained through self-cultivation in agricultural land and home yards. The respondents stated that they only found a small portion of the edible local fruits in the forest. Some fruits could be found on the seafront, grown wild and from outside collectors. In North Minahasa and Southeast Minahasa, the fruits were available in their own farmland and also in the yard (Fig. 11).

**Fig. 12:**
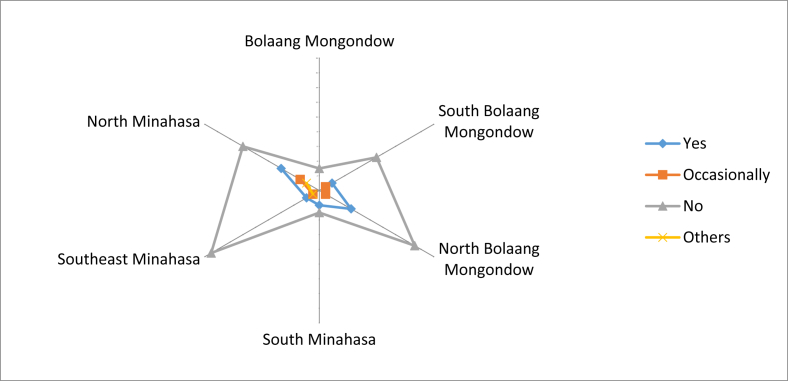
Medicinal preparations of the local edible fruit.

**Fig. 13:**
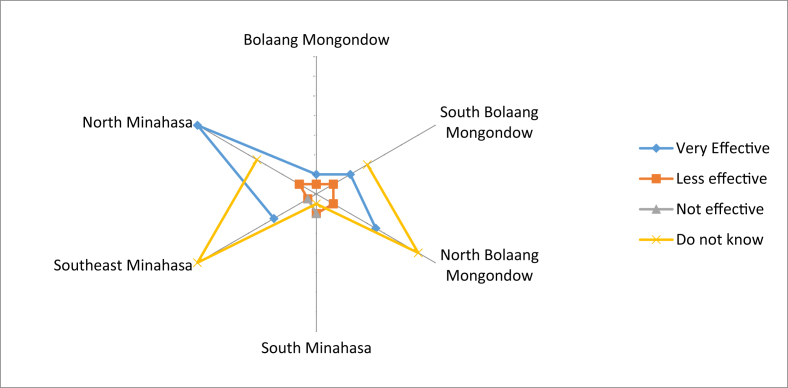
The effectiveness of local edible fruit as medicine.

Overall, most local fruits that were used as medicine were not made as preparations by the community. Only a small portion of the community made these preparations by themselves. People in Bolaang Mongondow did not process the fruits into medicinal preparations (Fig. 12). Many respondents in Southeast Minahasa and North Bolaang Mongondow did not know about the effectivity of these fruits as medicine. However, there were also many of them who claimed to have received benefits from the fruits, as found in North Minahasa (Fig. 13).

**Fig. 14:**
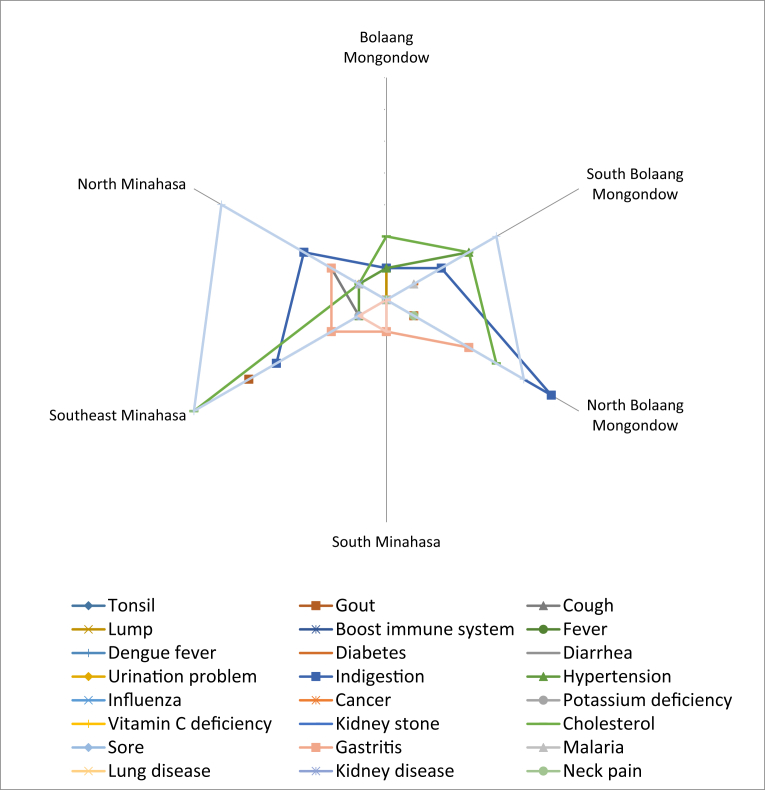
Types of diseases suffered by the community and can be cured by local fruits.

Types of diseases that were often suffered by people and cured by using local edible fruits were sprue, high cholesterol and digestive disorders. Sprue was cured most often using local fruits in Southeast Minahasa, North Minahasa, North Bolaang Mongondow, and South Bolaang Mongondow. Local fruit used to reduce cholesterol were mostly by the Minahasan. The digestive disorder was claimed to be cured mostly by the community in North Minahasa, Southeast Minahasa, and North Bolaang Mongondow (Fig. 14).

**Fig. 15:**
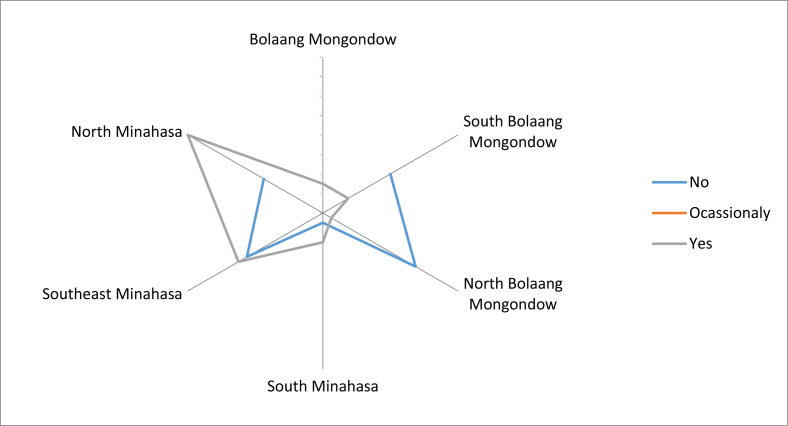
The way people consume the local fruits.

In addition to being eaten raw, some local fruits were further processed, for example made as juice, salad, compote, or mixed with food seasonings such as soy sauce, brown sugar, and made as vegetables. North Minahasa and Southeast Minahasa were where the community mostly consumed the processed fruits. Respondents from South Bolaang Mongondow, North Bolaang Mongondow, and some of respondents from Southeast Minahasa preferred to eat raw fruits (Fig. 15).

**Fig. 16:**
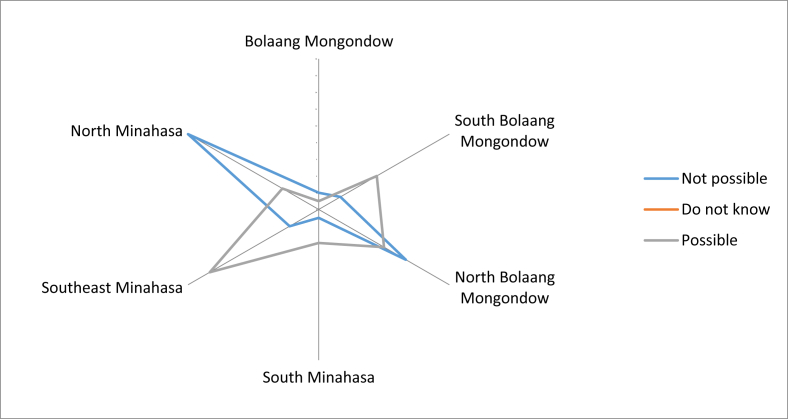
The possibility of young people who will be reluctant to consume local fruits.

The possibility of the younger generation who would be reluctant to consume these fruits was very high, according to most respondents in Southeast Minahasa, South and North Bolaang Mongondow. However, respondents from North Minahasa and some from North Bolaang Mongondow were still optimistic that the younger generation still wanted to consume local edible fruits (Fig. 16). However, they were still concerned that the younger generation would prefer imported fruits in the future. The importance of the local fruit conservation was realized by the respondents, however but they did not have enough knowledge about local fruit conservation practices, other than what they had done so far.

## 2 Experimental design, materials and methods

**Fig. 17:**
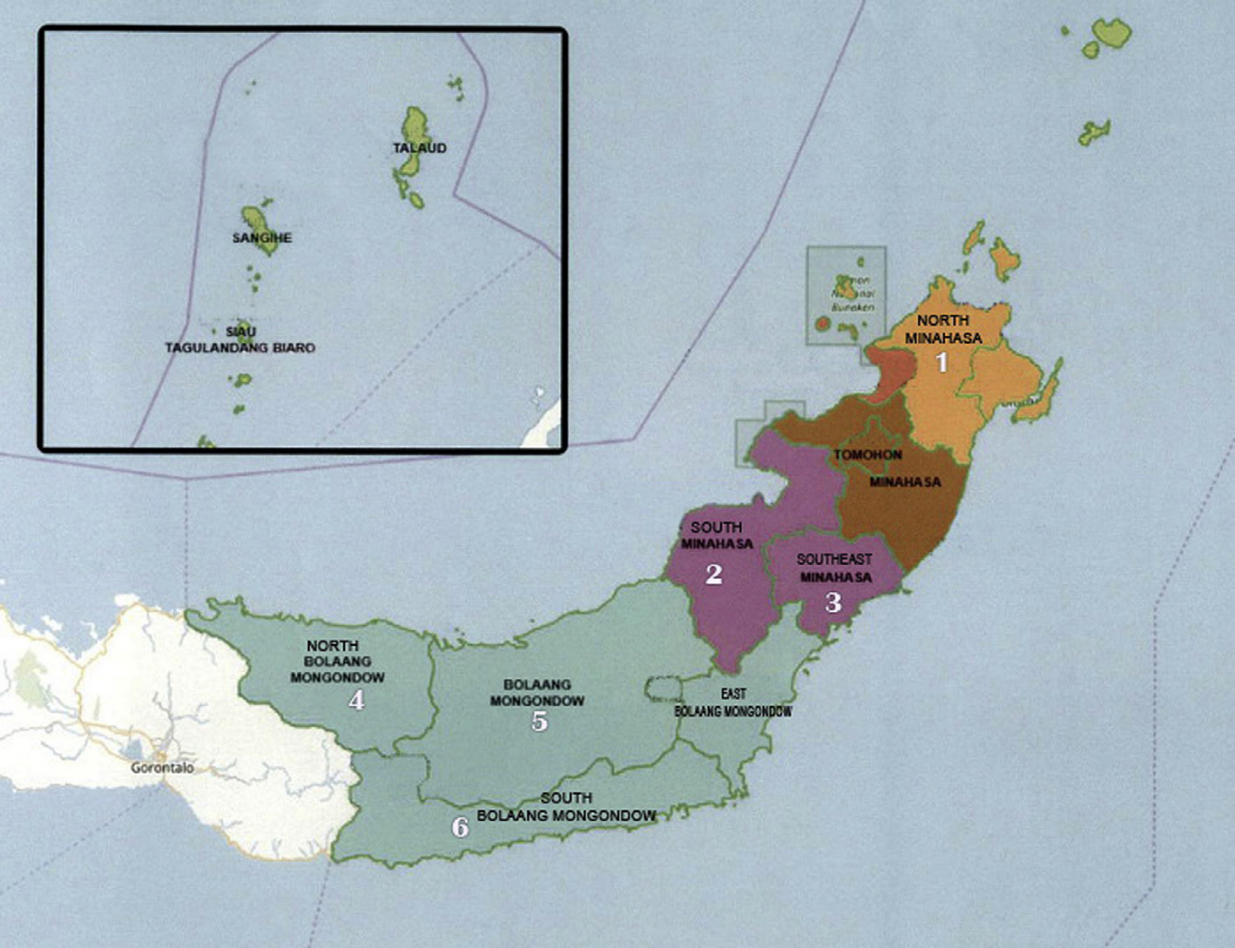
The map of North Sulawesi showing 6 regencies where data were collected.

Fieldwork was conducted in six regencies in North Sulawesi: North Minahasa [1], South Minahasa [2], Southeast Minahasa [3], North Bolaang Mongondow [4], Bolaang Mongondow (5), and South Bolaang Mongondow (6) (Fig. 17) between March and June 2019. The Code of Ethics of the International Society of Ethnobiology protocol (2006)[4] was followed to conduct the survey. The collection of ethnobotanical data was conducted through semi-structured and structured interviews as well as questionnaire to the key respondents on the existence, the use, and the conservation practices of local edible fruits. The lists of questionnaires and interviews are available at DOI: https://doi.org/10.17632/h4z52vfhmc.1#folder-f3f8a5a4-e0d7-4c05-8432-a3a55e22b635.

Key respondents were people who were able to provide accurate information, had good knowledge about the environment and the diversity of the edible local fruits. Criteria for selecting key respondents included local people who had lived in each regency for a minimum of 20 years, were more than 35 years old and knew or had used the fruits. The reason for selecting informants with the above criteria, so that the data obtained were accurate because the informants were aware of the local edible fruits in their area.
